# Management of Hepatitis B Virus Reactivation in Malignant Lymphoma Prior to Immunosuppressive Treatment

**DOI:** 10.3390/jpm11040267

**Published:** 2021-04-02

**Authors:** Yu-Fen Tsai, Chin-Mu Hsu, Hui-Hua Hsiao

**Affiliations:** 1 Department of Hematology & Oncology, E-Da Cancer Hospital, Kaohsiung 824, Taiwan; 970384kmuh@gmail.com; 2 School of Chinese Medicine for Post Baccalaureate, College of Medicine, I-Shou University, Kaohsiung 824, Taiwan; 3 Division of Hematology and Oncology, Department of Internal Medicine, Kaohsiung Medical University Hospital, Kaohsiung 807, Taiwan; e12013@gmail.com; 4 Center for Cancer Research, Kaohsiung Medical University, Kaohsiung 807, Taiwan; 5 Center for Liquid Biopsy and Cohort Research, Kaohsiung Medical University, Kaohsiung 807, Taiwan; 6 Cancer Center, Kaohsiung Medical University Hospital, Kaohsiung Medical University, Kaohsiung 807, Taiwan; 7 Faculty of Medicine, Kaohsiung Medical University, Kaohsiung 807, Taiwan

**Keywords:** hepatitis B reactivation, lymphoma, immunosuppressive therapy, nucleoside analogues

## Abstract

Hepatitis B reactivation is a common complication in lymphoma patients under immunosuppressive treatment with potentially serious and life-threating consequences. In this review, we discuss the basis of chronic Hepatitis B virus (HBV) infection, the definition and risk factors for HBV reactivation. We overview the management of HBV reactivation based on virological status and immunosuppressive regimen risk stratification. We also highlight and update information about the HBV reactivation in lymphoma patients under novel agent treatment, including newer monoclonal antibodies, small molecule inhibitors, and even chimeric antigen receptor T-cell immunotherapy.

## 1. Introduction

Hepatitis B virus (HBV) is an enveloped DNA virus that can cause a potentially life- threatening liver disease, such as liver failure and/or hepatocellular carcinoma. Although HBV vaccination and effective drugs for suppression of HBV have been widely applied in world, HBV infection is still a global health problem. Approximately 2 billion people have serological evidence of either past or present HBV infection and around 240 million people are chronically infected [1]. Chronic HBV infection is defined by the presence of HBsAg in serum for more than 6 months [2]. Chronic HBV infection is a dynamic equilibrium between HBV replication and the host immune system. The natural history of chronic HBV infection is classified into five phases: HBeAg-positive chronic HBV infection, HBeAg-positive chronic hepatitis B, HBeAg-negative chronic hepatitis B, HBeAg-negative chronic HBV infection, and HBsAg-negative phase, according to HBeAg, HBsAg, HBV DNA level, and alanine aminotransferase (ALT) value (Table 1) [2,3].

Hepatitis B reactivation mostly occurs in the context of an immunosuppressed status and has been commonly reported in cancer patients receiving chemotherapy or target therapy. HBV reactivation will cause significant morbidity and mortality if not appropriately diagnosed and managed. Clinicians should be aware of HBV reactivation and screen for HBV before implementing an immunosuppressive regimen and keep monitoring HBV status in high-risk population.

HBV reactivation has been an identified risk in lymphoma patients treated with cytotoxic chemotherapies (e.g., anthracyclines), high-dose corticosteroids, and anti-CD20 monoclonal antibody, rituximab. More and more novel agents, such as anti-CD30 monoclonal antibody, anti-CD52 monoclonal antibody, and small molecular inhibitors targeting Bruton tyrosine kinase (BTK), B-cell lymphoma-2 (BCL-2), phosphoinositide 3-kinase (PI3K), and even chimeric antigen receptor (CAR) T-cell immunotherapy have been used to treat malignant lymphoma in recent years. However, the risk of HBV reactivation in these novel agents is still undetermined. Thus, in this review, we will focus on the management of HBV reactivation in malignant lymphoma before immunosuppressive treatment and give a comprehensive and updated overview about the risk of HBV reactivation in the era of novel agents used to treat malignant lymphoma.

## 2. Definition of HBV Reactivation

HBV reactivation indicates the recurrence of active inflammatory disease in patients in the inactive phase of chronic hepatitis B (CHB) or those recovered from past infection. The definition of HBV reactivation varies between different guidelines, but the general principle is similar. In patients with CHB, HBsAg-positive at least 6 months, the reactivation is defined by an increase in HBV DNA level compared to baseline. In patients with resolved HBV infection, HBsAg-negative and anti-HBc-positive, reactivation is defined by the detection of HBV DNA or reappearance of HBsAg. Table 2 is the summary of definitions of HBV reactivation based on different society guidelines. A hepatitis flare is defined as ≥3-fold increase in ALT level compared to baseline and >100 U/L [2].

## 3. Risk Factors for HBV Reactivation

Multiple factors predispose HBV reactivation among patients who undergo immunosuppressive treatments. These risk factors can be divided into three parts: the host factors, virological factors, and immunosuppression regimens.

### 3.1. Host Factors

In previous studies, older age and male sex have been identified as risk factors for HBV reactivation [10,11,12,13]. Underlying disease needing immunosuppression therapy is also an important factor for HBV reactivation. However, the data are not so clear because it is difficult to distinguish the contribution of the underlying disease from that of the treatment used. Lymphoma is the common underlying disease acquiring HBV reactivation [14]. In addition, a survey of 1692 patients with hematologic malignancy found that diabetes mellitus, liver cirrhosis, and hepatocellular carcinoma were independent risk factors of HBV reactivation [15]. All these findings indicate that the immunocompromised host is a significant risk factor for HBV reactivation.

### 3.2. Virological Factors

HBV infection causes covalently closed circular DNA (cccDNA) in hepatocytes, regardless of HBsAg or HBV DNA status. The cccDNA is quite stable in infected cells and can persist in a latent state. The persistence of cccDNA is the key driver for HBV reactivation [16]. HBsAg positivity, HBeAg positivity and the high HBV DNA levels before immunosuppressive therapy have also been known risk factors for HBV reactivation [14,17,18,19]. The risk of HBV reactivation is five- to eight-fold higher in HBsAg patients [19]. High HBV viral load prior to cytotoxic chemotherapy is a prominently predictive factor for HBV reactivation [18]. In resolved HBV infection patients, many studies showed that negative baseline anti-HBs carried a higher risk of HBV reactivation [12,13,20,21,22,23]. In addition, mutation of HBsAg may also be a new issue of HBV reactivation. In a previous study, 93 patients had their HBsAg genetic features analyzed. Among them, 29 patients developed HBV reactivation and 75.9% of HBV reactivated patients carried HBsAg mutations localized in immune-active HBsAg region. These mutations enhanced had capability to evade immune response and were more susceptible to HBV reactivation [24].

### 3.3. Immunosuppressive Regimens

Different immunosuppressive drugs are characterized by different risks in HBV reactivation. The American gastroenterological association institute guideline classifies the immunosuppressive agents into high (>10% risk), moderate (1–10% risk), and low risk (<1% risk) groups for HBV reactivation [6,25]. Here, we modify the risk groups only for lymphoma patients and they are presented in Table 3. In addition, we also review specific immunosuppressive drugs, targets and novel agents treated in malignant lymphoma.

#### 3.3.1. Corticosteroids

Corticosteroids are used in the treatment of lymphoma for a long time. Firstly, corticosteroids upregulate the HBV glucocorticoid responsive element (a transcriptional regulatory element) and promote viral replications. Secondly, they cause a direct suppressive effect on cytotoxic T cells which is involved in HBV control [34]. The above two mechanisms explain the susceptibility to HBV reactivation in patients who receive corticosteroids. The immunosuppressive effect of steroids is dose and duration dependent. Guidelines from the American Gastroenterological Association (AGA) in 2015 present the high risk of HBV reactivation in HBsAg-positive patients treated with moderate (prednisolone 10–20 mg/day) or high dose (prednisolone > 20 mg/day) corticosteroid for ≥4 weeks. The following groups are at moderate risk of HBV reactivation: low dose (<10 mg prednisolone) corticosteroid used for ≥4 weeks in HBsAg-positive patients; moderate or high dose corticosteroid for ≥4 weeks in resolved HBV infection. Patients treated with corticosteroid <1 week at any dose are low risk of HBV reactivation [6]. In lymphoma patients, corticosteroids are usually used in combination with chemotherapy agents and an additive effect to increase HBV reactivation is noticed. In one randomized study, a total of 50 HBsAg-positive lymphoma patients were enrolled and treated with the same chemotherapy either without or with corticosteroid. The cumulative incidence of HBV reactivation at 9 months was significantly higher in the corticosteroid group (38% versus 73%, respectively, *p* = 0.03) [35].

#### 3.3.2. Anti-CD20 Monoclonal Antibodies

Rituximab, an anti-CD20 monoclonal antibody widely used in lymphoma treatment, has been well known a vital risk factor for HBV reactivation. Several studies have reported the risk of HBV reactivation in rituximab treatment. Both HBsAg-positive and resolved HBV infection patients who receive these agents are susceptible to HBV reactivation [13,20,36,37,38,39,40,41]. One study conducted in 104 diffuse large B-cell lymphoma patients treated either cyclophosphamide, doxorubicin, vincristine, and prednisone (CHOP) alone or rituximab plus CHOP (R-CHOP). Among HBsAg-negative/anti-HBc–positive diffuse large B-cell lymphoma (DLBCL) patients treated with R-CHOP, 25% (5/21) developed HBV reactivation; none had HBV reactivation in patients treated with CHOP [13]. Therefore, rituximab is a tremendous risk factor for HBV reactivation. The risk of HBV reactivation under these classes of drugs potentially persists for longer. Reactivation events have been reported up to two years after the last dose of rituximab. One prospective study of previous HBV exposure lymphoma patients without antiviral agent prophylaxis undergoing rituximab-containing chemotherapy reported a cumulative rate of HBV reactivation of 41.5% over two years. The time of HBV reactivation occurred at a median of 23 weeks (range, 4 to 100 weeks) after rituximab treatment [20]. Rituximab should not be avoided in case of HBV infection, as appropriate management of HBV is sufficient to prevent HBV reactivation.

Obinutuzumab, another new humanized monoclonal antibody to CD20, commonly used in the treatment of chronic lymphocytic leukemia (CLL), also increases HBV reactivation even in resolved HBV infection, similar with rituximab [42]. The mechanism of HBV reactivation in these drugs is causing depletion of circulating B cells and partial depletion of B cells in the lymphatic system and bone marrow. The risk of HBV reactivation persists for a longer period in B cell depleting agents than traditional chemotherapy. Delayed reactivation events (one year after withdrawal of antiviral prophylaxis or completion of immunosuppressive treatment) have been reported in several studies [43,44,45,46]. Therefore, prolonged monitoring of liver function and HBV titers for more than 1 year after immunosuppression is needed when giving this class of drugs.

#### 3.3.3. Other Monoclonal Antibodies

Alemtuzumab, a monoclonal antibody against CD52, used to treat refractory CLL or as a conditioning regimen in hematopoietic stem cell transplantation, has been reported to induce HBV reactivation and related hepatitis. Two CLL subjects with occult HBV infection developing a virological and biochemical flare of hepatitis B following immunotherapy with alemtuzumab were reported [47]. Another study reviewed 240 individuals with past exposure HBV receiving alemtuzumab-based reduced intensity conditioning bone marrow transplantation with lamivudine prophylaxis. Two HBV carrier recipients died of liver failure due to HBV reactivation and both occurred after stopping lamivudine at 8 months and 31 months post-bone marrow transplantation, respectively.

Brentuximab vedotin, an antibody–drug conjugate medication, which targets tumor cells expressing CD30, is used for T cell lymphoma and relapsed or refractory Hodgkin lymphoma. A case of HBV reactivation was reported from China. In total thirteen relapsed or refractory Hodgkin lymphoma patients were treated with brentuximab vedotin, and one occult HBV infection patient developed HBV reactivation [48]. Therefore, the risk of HBV reactivation in brentuximab vedotin should be concerned.

#### 3.3.4. Other Novel Agents

Ibrutinib is used to treat mantle cell lymphoma, chronic lymphocytic leukemia, Waldenstrom macroglobulinemia, marginal zone lymphoma and chronic graft-versus-host disease in allo-hematopoietic stem cell transplantation. It inhibits (Bruton’s tyrosine kinase (BTK) and thereby interrupts the B-cell receptor signaling pathway which regulates B cell proliferation and activation [49]. Blocking this pathway may suppress the immune control of HBV and induce HBV reactivation. Two case reports have described a fulminant HBV reactivation in CLL patients with past or occult HBV infection after ibrutinib treatment [30,31]. A retrospective study to evaluate the incidence of HBV reactivation among patients with past HBV infection and hematologic malignancy during and after ibrutinib therapy was conducted at the Dana-Farber Cancer Institute between 1 January 2010 and 31 December 2016 [32]. During the study period, 412 patients were treated with ibrutinib, and two of them developed HBV reactivation. This study concluded that HBV reactivation may be a risk in those with past HBV infection under ibrutinib treatment.

Idelalisib, a PI3K inhibitor, is approved for indolent lymphoma treatment. A total of 13–25% of patients reported grade 3 or higher elevations of serum aminotransferase levels and most cases are asymptomatic and resolved following dose reduction [50,51]. The risk of HBV reactivation in patients with idelalisib is still unclear currently.

Bortezomib, a proteasome inhibitor, is used for treatment of multiple myeloma (MM) and mantle cell lymphoma. A total of 139 patients with MM receiving bortezomib-containing regimens were enrolled. HBV reactivation occurred in six HBsAg-positive patients and two HBsAg -negative patients, including six who received autologous stem cell transplant (ASCT). Overall survival and progression free survival are both longer in HBsAg-negative patients, especially those who underwent ASCT [33]. Therefore, HBV prophylaxis is recommended for all HBsAg-positive patients who were treated with bortezomib.

Venetoclax is a potent inhibitor of the antiapoptotic BCL-2 protein and is used for CLL and acute myeloid leukemia treatment. The most common grade 1 or 2 adverse events were self-limited diarrhea and nausea. Neutropenia was the most common grade 3 or 4 adverse event and the most common serious adverse event was febrile neutropenia. No hepatic toxicity was reported [52,53]. It may have a potential risk of HBV reactivation, but no case has been reported to date. Thus, the real risk of HBV reactivation is still unknown for venetoclax.

Due to the lack of large retrospective or prospective studies on the HBV reactivation risk in novel agents, the definite incidence of HBV reaction is unclear. However, in view of the mechanisms of these novel agents, all the novel agents will suppress B cell activity. Thus, in our opinion, HBV prophylaxis should be given for HBsAg-positive or resolved HBV infection with detectable HBV DNA lymphoma patients when treated with these novel agents. For those with negative HBV DNA, resolved HBV infection patients, closely monitoring liver function and HBV DNA levels every 1 to 3 months is recommended. Initiating pre-emptive treatment with anti-viral agents when HBV DNA and/or liver function levels rise is recommended.

#### 3.3.5. Chimeric Antigen Receptor (CAR) T-Cell Immunotherapy

CAR T cell immunotherapy has recently been found to be a novel and effective treatment for relapsed or refractory diffuse large B-cell lymphoma (DLBCL) [54,55,56,57]. A case of severely early HBV reactivation in an inactive HBV carrier who was treated with the sequential infusion of anti-CD 19 and anti-CD 22 CAR T cells for relapsed and refractory DLBCL was reported [27]. The patient discontinued her antiviral prophylaxis by herself one month after CAR T cell therapy and severe HBV reactivation-related hepatitis happened. She finally passed away because of hepatic encephalopathy and multiple organ failure. This is the first report of severe HBV reactivation after CAR T cell therapy. More data are needed to define the incidence of HBV reactivation and duration of prophylaxis during CAR T cell therapy. However, HBV reactivation seems to be a significant complication in CAR T cell treatment and should be cautious. Therefore, prophylaxis is suggested for both HBsAg-positive and resolved HBV infection lymphoma patients.

## 4. Screening and Management of HBV Reactivation in Lymphoma Patient Prior Immunosuppression Therapy

Table 4 summarizes the different society recommendations about the screening and management of HBV reactivation in lymphoma patients prior to immunosuppression therapy.

### 4.1. Screening

All patients undergoing immunosuppressive therapy should be screened for HBV infection before the initiation of treatment. There are few variations about screening tests between different guidelines (Table 4). Most guidelines recommend screening tests including HBsAg and anti-HBcAb. A serum HBV DNA test should be carried our if either positive for HBsAg or anti-HBcAb.

### 4.2. Strategies for HBV Reactivation

Strategies for HBV reactivation can be prophylaxis or pre-emptive (=on demand) therapy; the former strategy offers antiviral agent to patients prior to immunosuppressive therapy, whereas the latter commence regular monitoring (every 1–3 months) of HBsAg, ALT and HBV DNA during immunosuppressive therapy and starting antiviral agent only when HBsAg, ALT and/or HBV DNA levels rise.

To select the population who require prophylactic antiviral therapy, we need to assess the risk factors for reactivation, as mentioned earlier. The details of virological markers and immunosuppressive regimens should be carefully evaluated individually. When anti-HBV prophylaxis is indicated, antiviral agent is recommended for administration before 7 days before the initiation of immunosuppressive therapy.

#### 4.2.1. HBsAg-Positive Patients without Hepatitis at Baseline (Inactive Phase of CHB)

Studies have shown that a preventive strategy is more effective in preventing HBV reactivation in HBsAg-positive patients [58,59]. All HBsAg-positive candidates regardless of their HBV DNA are recommended to accept nucleotide analogues (NAs) for prophylaxis prior immunosuppressive therapy.

#### 4.2.2. HBsAg-Negative and Anti-HBcAb-Positive Patients

The risk of HBV reactivation in this group varies widely according to the viremia condition, underlying disease and the types of immunosuppression regimens. These subjects are suggested to test HBV DNA. If HBV DNA is detectable, they should be treated similarly to HBsAg-positive patients. If HBV DNA is undetectable, the next step is to assess the immunosuppressive regimen risk stratification (Table 2). High risk groups (e.g., anti-CD20 monoclonal antibody) (>10%) should be receive antiviral prophylaxis with NAs. Huang et al. compared prophylactic entecavir treatment and pre-emptive entecavir treatment in lymphoma patients with resolved hepatitis B who received rituximab–CHOP (rituximab–cyclophosphamide, doxorubicin, vincristine, and prednisolone) chemotherapy, and confirmed that prophylactic entecavir treatment significantly reduced the risk of HBV reactivation (17.9% versus 2.4%, *p* = 0.027) [44]. In moderate (<10%) or low (<1%) risk groups, prophylaxis is not routinely suggested and periodical monitoring of HBsAg and HBV DNA level at intervals of 1 to 3 months is generally recommended. Pre-emptive treatment by initiating NAs when HBV DNA and/or ALT levels rise is recommended.

#### 4.2.3. HBsAg and Anti-HBcAb-Negative Patients

Since some studies found that anti-HBs potentially decreased the risk of HBV reactivation [21,22,23], HBV vaccination should be considered in HBsAg-negative and anti-HBc-negative patients without anti-HBs population [3].

### 4.3. Choices of Antiviral Agents

Interferons (IFNs) and NAs are used to treat chronic HBV infection. Since NAs have less severe adverse reactions and a higher barrier to antiviral resistance, NAs are the preferred choices on the treatment of chronic HBV infection. There are six types of NAs approved for chronic HBV treatment: lamivudine (LMV), adefovir (ADV), telbivudine (LdT), entecavir (ETV), tenofovir (TDF), and tenofovir alafenamide fumarate (TAF). Multiple studies and meta-analyses have demonstrated that entecavir or tenofovir are the preferred first line antiviral agents because of their higher potency and high resistance barrier [29,60,61,62]. In a randomized controlled trial, the efficacy of entecavir versus lamivudine was compared in diffuse large B cell lymphoma patients with HBsAg-positive undergoing R-CHOP treatment. The study found that entecavir is more effective than lamivudine in reducing the risk of HBV reactivation (6.6% versus 30%), and HBV-related hepatitis (0% versus 13.3%) [62]. Therefore, several guidelines on the management of HBV infection including the American, European and Asian guidelines suggest ETV, TDF, or TAF as the first line agents for HBV prophylaxis and treatment [2,3,7,63,64,65]. However, lamivudine may be considered for anti-HBc-positive patients requiring a short duration of therapy (<6 months) with low and intermediate risk of immunosuppression [66].

### 4.4. Duration of Antiviral Agents

The exact duration of antiviral agents remains controversial. Generally antiviral prophylaxis for chronic HBV, inactive phase and resolved HBV infection should last for a minimum of 6 to 12 months after completion of chemotherapy; in the case of rituximab and B cell depleting agents, the recommendation of prophylaxis is at least 12 months after completion of treatment [2,7,8,25,64]. However, delayed HBV reactivation has been reported for patients who received anti-CD20 antibody therapy. Thus, EASL guidelines suggest extending the duration to more than 12 months and for up to 18 months [3,63,67].

### 4.5. Duration of Monitoring after Cessation of Antiviral Agents

Periodic monitoring after antiviral agents is recommended. Generally, liver function tests, HBsAg, and HBV DNA suggest continuing monitoring for at least 12 months after discontinuation of antiviral agents; for those receiving anti-CD20 antibody therapy, monitoring for more than 12 months is recommended. The interval of monitoring is considered every 3–6 months [2,3,9].

## 5. Summary

**Figure 1:**
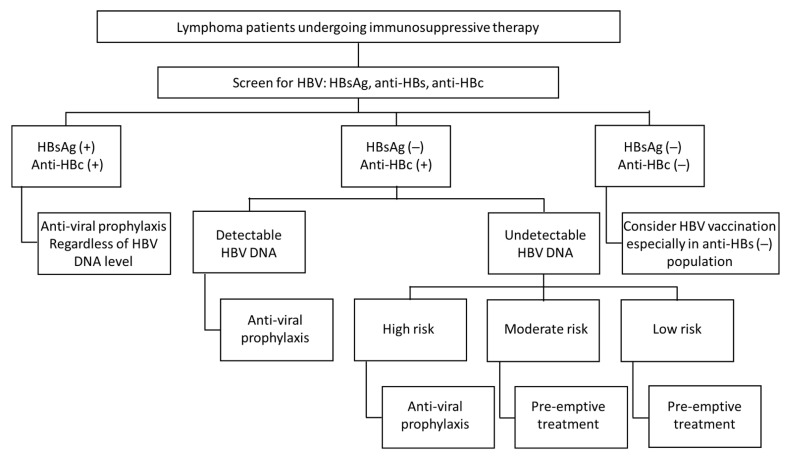
Algorithm for the screening and management of HBV reactivation in lymphoma patients undergoing immunosuppressive therapy.

The algorithm for screening and management of HBV reactivation in lymphoma patients undergoing immunosuppressive treatment is adapted from different guidelines, reviews [2,3,8,25,37,67,68], and personal opinions and is shown in Figure 1.

## 6. Conclusions

HBV reactivation exerts a negative impact on the clinical outcomes in patients with malignant lymphoma. All patients should be screened for HBV status prior immunosuppressive therapy. The risk of HBV reactivation should be assessed by virological markers and immunosuppressive regimens individually. Given the efficacy of NAs against HBV, prophylactic therapy is a more effective strategy to manage HBV reactivation. Prophylaxis is recommended for all HBsAg-positive patients and HBsAg-negative, anti-HBc-positive patients with detectable HBV DNA or high risk for HBV reactivation. Liver function, HBsAg, and HBV DNA should continue to be monitored for at least 12 months after the cessation of prophylaxis. Several novel agents for lymphoma treatment have emerged in recent years. However, only few HBV reactivation cases were reported. The real incidence and risk of HBV reactivation in these novel agents is still unclear to date. It is imperative to conduct larger studies to clarify the risk of HBV reactivation and provide a comprehensive guideline to prevent HBV reactivation during novel agent treatment.

## Figures and Tables

**Table 1:** Natural course of chronic hepatitis B *.

Phase	1	2	3	4	5
	HBeAg-positive chronic infection, also called Immune tolerant phase	HBeAg-positive chronic hepatitis, also called immune reactive phase	HBeAg-negative chronic hepatitis	HBeAg-negative chronic infection, also called inactive carrier phase	HBsAg-negative, also called resolved HBV infection or occult HBV infection ^a^
Serological marker	HBeAg (+); Anti-HBe (−)	HBeAg (+); may develop anti-HBe	HBeAg (−); Anti-HBe (+/−)	HBeAg (−); Anti-HBe (+)	HBsAg (−); Anti-HBc (+) ^a^; Anti-HBs (+/−)
HBV DNA	very high levels, generally ≥10^7^ IU/mL	10^4^–10^7^ IU/mL	>2000 IU/mL	Generally <2000 IU/mL or negative	<200 IU/mL or negative
ALT	Normal	Elevated	Elevated	Normal	Normal
Liver disease	None/Minimal	Moderate/severe	Moderate/severe	None/minimal	None

* Reference: [2,3]. ^a^: Occult Hepatitis B virus (HBV) infection (OBI) can be defined as the long-lasting persistence of viral genomes in the liver tissue (and in some cases also in the serum) [4]. Based on HBV specific antibodies, there are two groups of OBI: seropositive OBI: anti-HBcAb and/or anti-HBsAb positive; seronegative OBI: anti-HBcAb and anti-HBsAb-negative [5].

**Table 2:** Definitions of HBV reactivation based on different society guidelines.

Society	Reactivaion of CHB	Reactivation of Resolved HBV
American Association for the Study of Liver Diseases (AASLD) 2018 guideline [2]	Any of the following: Unavailable DNA baseline: ≥10,000 IU/mL Available DNA baseline, previously undetectable: ≥1000 IU/mL Available DNA baseline, previously detectable: ≥100-fold increase	Any of the following: Development of detectable DNA Reappearance of HBsAg (also known as reverse seroconversion)
American Gastroenterological Association (AGA) 2015 guideline [6]	Unavailable DNA baseline: not explicitly defined Available DNA baseline, previously undetectable: de novo detectable DNA Available DNA baseline, previously detectable: ≥10-fold increase	Reverse seroconversion to HBsAg-positive status
The Asian Pacific Association for the Study of the Liver (APASL) 2016 guideline [7]	Unavailable DNA baseline: ≥20,000 IU/mL Available DNA baseline, previously undetectable: de novo detectable HBV DNA to a level of 100 IU/mL Available DNA baseline, previously detectable: ≥2 log increase from baseline levels	Not clearly defined
European Association for the Study of the Liver (EASL) 2017 guideline [3]	No clearly defined	Not clearly defined
Korean Association for the Study of the Liver (KASL) 2019 guideline [8]	An increase in serum HBV DNA by more than 100 times the baseline level	Seroconversion of HBsAg-negative to positive Detection of serum HBV DNA from none to positive
American Society of Clinical Oncology (ASCO) 2020 update [9]	The same as the AASLD guidelines	The same as the AASLD guidelines

**Table 3:** Risk groups based on immunosuppressive agents treated in lymphoma patients (adapted from the American Gastroenterological Association (AGA) guideline [6]).

Risk Group	Immunosuppressive Agents	
	HBsAg-Positive, Anti-HBc-Positive	HBsAg-Negative, Anti-HBc-Positive
High risk (>10%)	B-cell depleting agents: rituximab, obinutuzumab, alemtuzumab Anthracycline derivatives: doxorubicin, epirubicin High dose corticosteroids (≥20 mg prednisolone for ≥4 weeks) Stem cell transplantation [26] Chimeric antigen receptor (CAR) T-cell immunotherapy [27] *	B-cell depleting agents: rituximab, obinutuzumab, alemtuzumab Stem cell transplantation [28] Chimeric antigen receptor (CAR) T-cell immunotherapy [29] *
Moderate risk (1–10%)	Systemic chemotherapy Tyrosine kinase inhibitors: ibrutinib [30,31,32] * Proteasome inhibitors, such as bortezomib [33] * Corticosteroid therapy for ≥4 weeks regardless of dosage	Tyrosine kinase inhibitors: ibrutinib [30,31,32] * Proteasome inhibitors, such as bortezomib [33] * Corticosteroid (≥10 mg prednisolone for ≥4 weeks) Systemic chemotherapy including anthracycline derivatives: doxorubicin, epirubicin
Low risk (<1%)	Corticosteroid therapy for ≤1 week Methotrexate	Corticosteroid therapy for ≤1 week Corticosteroid therapy < 10 mg prednisolone for ≥4 weeks Methotrexate

* We modified the table and added some novel agents and therapies to the table based on case reports, significant complications after HBV reactivation and personal opinions.

**Table 4:** Guidelines on screening and management for HBV reactivation before immunosuppression therapy.

Society	Who Should Be Screened?	Screening Tests	Strategy	Choice of NAs	NAs Duration	Monitoring after Prophylaxis
American Association for the Study of Liver Diseases (AASLD) 2018 guideline [2]	All patients	HBsAg and anti-HBcAb	HBsAg (+): prophylaxis Resolved HBV: on- demend therapy except for patients receiving anti-CD20 antibody therapy or stem cell transplantation (monitor ALT, HBV DNA, HBsAg every 1–3 months)	ETV, TDF, TAF	At least 6 months after discontinuation of immunosuppressive therapy At least 12 months for B cell–depleting agents	Patients should be monitored for up to 12 months after cessation of anti-HBV therapy
American Gastroenterological Association (AGA) 2015 guideline [25]	Moderate or high risk of HBV reactivation	HBsAg and anti-HBc, HBV DNA test if either positive	High and moderate risk: prophylaxis Low risk: against routine prophylaxis	Antivirals with highbarrier to resistance over lamivudine	At least 6 months after discontinuation of immunosuppressive therapy At least 12 months for B cell–depleting agents	Not mentioned
The Asian Pacific Association for the Study of the Liver (APASL) 2016 guideline [7]	All patients	HBsAg and anti-HBcAb, HBsAg (−), anti-HbcAb (+): HBV DNA	HBsAg (+): prophylaxis Resolved HBV with detectable HBV DNA: treated as HBsAg (+) Resolved HBV with undetectable HBV DNA: on demand therapy, except anti-CD20 antibody therapy or stem cell transplantation (monitor ALT and HBV DNA every 1–3 months)	ETV, TDF	At least 12 months after cessation of therapy	Not mentioned
European Association for the Study of the Liver (EASL) 2017 guideline [3]	All patients	HBsAg, anti-HbcAb, and anti-HbsAb	HBsAg (+): prophylaxis Resolved HBV, high risk: prophylaxis Resolved HBV, moderate and low risk: on- demend therapy (monitor HBsAg and/or HBV DNA every 1–3 months)	ETV, TDF, TAF	At least 12 months after cessation of the immunosuppressive treatment At least 18 months for rituximab-based regimens	Liver function tests and HBV DNA should be tested every 3 to 6 months and for at least 12 months after NAs withdrawal
Korean Association for the Study of the Liver (KASL) 2019 guideline [8]	All patients	HBsAg and anti-HbcAb, HBV DNA test if either positive	HBsAg (+) or HBV DNA detectable: prophylaxis Resolved HBV anti-CD20 antibody therapy or stem cell transplantation: prophylaxis Resolved HBV moderate or low risk: on- demend therapy (monitor HBsAg and HBV DNA every 1–3 months)	ETV, TDF	At least 6 months after the chemotherapy is completed At least 12 months for rituximab-based regimens after the completion of chemotherapy	Not mentioned
American Society of Clinical Oncology (ASCO) 2020 update [9]	All patients	HBsAg, anti-HBcAb, and anti-HBsAb	HBsAg (+): prophylaxis Resolved HBV, high risk, e.g., anti-CD20 antibody therapy or stem cell transplantation: prophylaxis Resolved HBV, other therapy: on-demend therapy (monitor HBsAg and HBV DNA every 3 months)	ETV, TDF, TAF	At least 12 months after cessation of the immunosuppressive treatment	HBsAg (+) and resolved HBV with high risk: monthly for the first 3 months after NAs withdrawal and then every 3 months, no comment on duration Resolved HBV, no high risk: not necessary

ETV: entecavir, HBV: hepatitis B virus, NAs: nucleotide analogues, TDF: tenofovir, TAF: Tenofovir alafenamide fumarate.

## Data Availability

Not applicable.
